# Gestational diabetes mellitus and linear growth in early childhood

**DOI:** 10.3389/fendo.2024.1470678

**Published:** 2024-11-27

**Authors:** Zi-Lin Chen, Xin Liu, Min-Yi Tao, Meng-Nan Yang, Hua He, Fang Fang, Ting Wu, Fengxiu Ouyang, Jun Zhang, Fei Li, Zhong-Cheng Luo

**Affiliations:** ^1^ Ministry of Education-Shanghai Key Laboratory of Children’s Environmental Health, Early Life Health Institute, and Department of Pediatrics, Xinhua Hospital, Shanghai Jiao-Tong University School of Medicine, Shanghai, China; ^2^ Department of Obstetrics and Gynecology, Mount Sinai Hospital, and Institute of Health Policy, Management and Evaluation, Temerty Faculty of Medicine, University of Toronto, Toronto, ON, Canada; ^3^ Chengdu Women’s and Children’s Central Hospital, School of Medicine, University of Electronic Science and Technology of China, Chengdu, China

**Keywords:** early childhood, gestational diabetes mellitus, linear growth, length/height for age z score, birth cohort

## Abstract

**Introduction:**

Gestational diabetes mellitus (GDM) is a common pregnancy complication with potential short- and long-term adverse consequences for both mothers and fetuses. It is unclear whether GDM affects linear growth in the offspring; research data are limited and inconsistent.

**Methods:**

In a prospective birth cohort in Shanghai (n=2055 children; 369 born to mothers with GDM). We sought to evaluate the impact of GDM on longitudinal linear growth in early childhood. Length/height was measured in children at birth, 6 weeks, 6 months, 1, 2 and 4 years of age. Multivariate linear regression and generalized estimating equation models were employed to assess the impact of GDM on length/height for age Z score (LAZ/HAZ).

**Results:**

Average birth length was similar in infants of GDM *vs*. euglycemic mothers. Adjusting for maternal and child characteristics, the children of mothers with GDM had consistently lower LAZ/HAZ compared to children of mothers without diabetes at ages 6 weeks, 6 months, 1, 2 and 4 years. GDM was associated with a 0.12 (95% confidence intervals 0.04-0.21) deficit in LAZ/HAZ in the growth trajectory from birth to age 4 years after adjusting for maternal and child characteristics.

**Discussion:**

GDM was associated with impaired longitudinal linear growth in early childhood. Further studies are warranted to understand the long-term impact on stature and health.

## Introduction

Gestational diabetes mellitus (GDM) is a common pregnancy complication characterized by *de novo* glucose intolerance during gestation affecting both the fetuses and the mothers (1, 2). GDM has been associated with macrocosmic birth and increased risks of obesity and glucose intolerance in the offspring in later life (3–5). It is unclear whether GDM affects linear growth in the offspring. Basic science studies indicate that the osteogenic capability of bone marrow mesenchymal stem cells is impaired under high glucose conditions (6, 7), suggesting that a high-glucose environment may affect bone’s growth potential. However, research data are limited and inconsistent concerning whether GDM affects linear growth in the offspring at birth (8–19), during infancy (0-2 years) (8, 9, 13, 14, 18–24), early childhood (2-5 years) (8, 13, 25–27), late childhood (6-10 years) (13, 28–30) and adolescence (11-18 years) (29, 31, 32). Some studies reported lower height or reduced linear growth in the offspring of mothers with GDM (9, 28, 31, 32), while others reported increased height (8, 13, 33) or no significant differences (12, 26, 27, 29). Common limitations in previous studies included inadequate sample sizes (18–20, 28), inaccurate length/height data based on routine childcare records rather than standardized measurements (13, 21, 27, 31), and lack of adjustment for major confounding factors (such as maternal height) (8, 13, 18, 19, 21, 22, 26). Longitudinal data are scanty concerning the impact of GDM on linear growth in early childhood. In the present study, we sought to assess the impact of GDM on longitudinal linear growth in early childhood (from birth to age 4 years) in a large prospective birth cohort.

## Methods

### Study design and population

This was a prospective follow-up study of children in the Shanghai Birth Cohort (SBC) (34). The SBC is a prospective cohort involving 4127 pregnancies in six tertiary obstetric care hospitals in Shanghai between 2013 and 2016. The study was approved by the research ethics boards of Shanghai Xinhua Hospital (the coordination center) and all participating hospitals. Written informed consent was obtained from all study participants.

**Figure 1:**
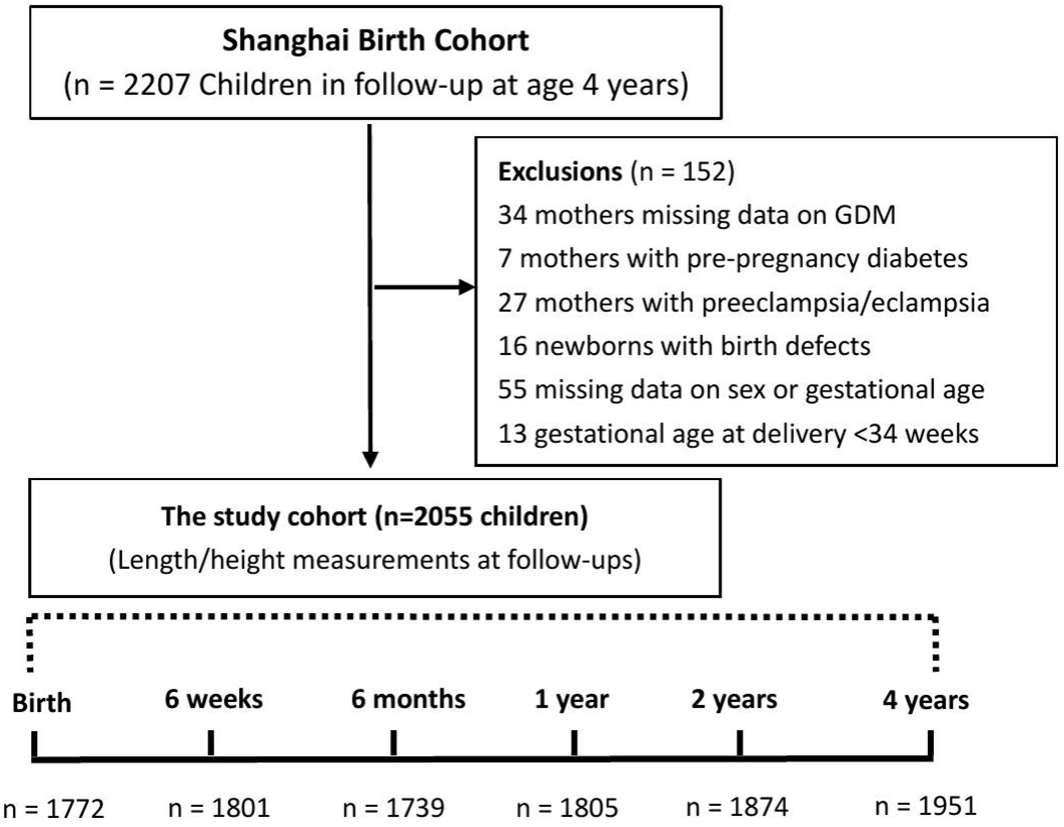
Flowchart in the selection of study subjects and child’s length/height measurements in the Shanghai Birth Cohort. GDM, gestational diabetes mellitus.

There was a total of 3692 live births (3331 singletons) in the SBC, and 2207 singleton children remained in follow-ups at age 4 years. We excluded children whose mothers had pre-gestational diabetes (n=7) or preeclampsia/eclampsia (n=27), and children with birth defects (n=16), born at gestational age <34 weeks (n=13), or missing information on maternal GDM (n=34), child’s sex or gestational age at delivery (n=55). The final study sample included 2055 children with at least one follow-up length/height measurement between ages 6 weeks and 4 years. The numbers of children with data available on length/height were 1772 (86%) at birth, 1801 (88%) at 6 weeks, 1739 (85%) at 6-months, 1805 (88%) at 12 months, 1874 (91%) at 2 years, and 1951 (95%) at 4 years of age, respectively. There were 1139 children with complete data on length/height measurements at all the six time points. **Figure 1** illustrates the selection of study subjects and follow-up length/height measurements from birth to 4 years of age.

GDM was diagnosed by a 75g oral glucose tolerance test at 24-28 weeks of gestation according to International Association of Diabetes and Pregnancy Study Groups (IADPSG)’ criteria (35): if any blood glucose value reaches or exceeds the following thresholds: fasting 5.1 mmol/L, 1- hour 10.0 mmol/L, and 2-hour 8.5 mmol/L. There were 369 mothers with GDM in the study cohort.

### Maternal/pregnancy characteristics

Available maternal/pregnancy characteristics included age, ethnicity, parity, education, smoking or alcohol use during pregnancy, hypertensive disorders in pregnancy (chronic or gestational, excluding preeclampsia or eclampsia), height, pre-pregnancy weight, gestational weight gain, mode of delivery, infant’s sex, birth weight and gestational age. Pre-pregnancy body mass index (BMI) was calculated as weight (kg)/(height [m]) ^2. Gestational weight gain was calculated as the difference between the weight measured within one week before delivery and pre-pregnancy weight, and was classified as inadequate, adequate, and excessive if it was below, within, or above the recommendations of the 2009 Institute of Medicine guidelines (36): 12.5-18.0 kg for underweight; 11.5-16.0 kg for normal weight; 7.0-11.5 kg for overweight; and 5.0-9.0 kg for obese women. Women were classified by pre-pregnancy BMI as underweight (<18.5 kg/m^2^), normal weight (18.5-24.9 kg/m^2^), overweight (25.0-29.9 kg/m^2^) or obese (30.0+ kg/m^2^). Gestational age was determined by the date of last menstruation period and confirmed by first-trimester ultrasound dating. If the difference between the 2 estimates was more than 2 weeks, the ultrasound dating-based estimate was used.

### Length/height measurements in children

Length/height was measured at birth and postnatal follow-ups at ages 6 weeks (42 days), 6 months, 1, 2 and 4 years by trained research staff. Length (birth to age 2 years) and standing height (age 4 years) were measured following standardized operating protocols. Length (birth to 2 years of age) were measured in supine position using a Seca 416 Infantometer (Seca Netherlands, Hamburg). At age 4 years, standing height was measured by a wall-mounted stadiometer (Seca Netherlands, Hamburg). Length and height measurements were measured twice to the closest 0.1 cm, and the averages were taken as the final values.

Length/height-for-age Z scores were calculated according to the World Health Organization (WHO) growth references (37) using the ‘anthro’ and ‘anthroplus’ package in R.

### Statistical analysis

Data are presented as mean ± SD for continuous variables or frequency (percentage) for categorical variables. The independent t-test and Pearson’s chi-square test were used to compare differences in continuous and categorical variables between GDM and euglycemic pregnancy groups, respectively. Generalized linear models were employed to evaluate the association between GDM and LAZ/HAZ at each follow-up time point. Generalized estimating equation (GEE) models were used to assess the association between GDM and linear growth trajectory from birth to 4 years of age overall accounting for the correlations in length/height measurements over ages within the same subjects. In the adjusted models, we included pre-specified known factors affecting length/height (maternal height, infant’s sex, gestational age at birth). Other co-variables, including maternal age, ethnicity, parity, smoking in pregnancy, alcohol drinking in pregnancy, pre-pregnancy BMI, gestational weight gain, hypertensive disorders in pregnancy, mode of delivery and breastfeeding were subject to a stepwise regression selection process. Only co-variables with P<=0.20 would be retained in the final parsimonious models.

Since pre-pregnancy BMI may be considered an upstream risk factor of GDM, gestational weight gain may be influenced by maternal GDM, and GDM may affect postnatal linear growth through affecting gestational age at birth, we repeated the stepwise regression models in sensitivity analyses excluding gestational age, or gestational weight gain, or both pre-pregnancy BMI and gestational weight gain. To disentangle the potential confounding effects of maternal hypertension from GDM in the associations with offspring’s linear growth, we excluded those exposed to maternal hypertensive disorders in a sensitivity analysis. Considering the potential impact of paternal height, we also conducted a sensitivity analysis to include paternal height (with a high frequency of missing values) in the adjusted models in a sensitivity analysis.

Effect sizes in regression coefficients (β) with 95% confidence intervals (CIs) were presented. Missing values in continuous co-variables were not imputed. Missing values in categorical co-variables were taken as a valid category in regression models. P<0.05 was considered statistically significant in assessing the primary association of interest between GDM and linear growth from birth to age 4 years in a GEE model. P values in other comparisons were for exploratory information only, and multiple comparisons were not accounted for. All statistical analyses were conducted using R version 4.2.3 and STATA V.15.

## Results

Of the 3331 singleton births in Shanghai birth cohort, 2207 children remained in follow-ups at age 4 years, 2055 children were included in the final study cohort (**Figure 1**). Comparing children who lost to *vs*. remained in follow-ups at age 4 years (**Supplementary Table S1**), their mothers tended to be younger (mean: 29.1 *vs*. 29.5 years), had higher pre-pregnancy weight (mean: 59.5 kg *vs*. 56.4 kg) and BMI (mean: 22.6 *vs*. 21.5 kg/m^2^) and were likely to have GDM (18.1% *vs*. 12.7%), while the children had shorter gestational age (mean: 38.9 *vs*. 39.1 weeks) and were more likely to be delivered by cesarean section (52.4% *vs*. 45.6%). Other maternal and infant characteristics were similar.

### Characteristics of study subjects

**Table 1** presents the characteristics of subjects in the final study cohort. The average age of mothers at delivery was 29 years, and all mothers were over 20 years of age. There were 369 mothers (18%) with GDM. Compared to mothers with a euglycemic pregnancy, mothers with GDM were older (mean: 30.2 *vs*. 28.7 years), less likely to have completed college or higher education, had higher pre-pregnancy BMI (mean: 24.1 *vs*. 22.2 kg/m2), but were less likely to have excessive weight gain during pregnancy (12.7% *vs*. 24.7%). GDM mothers were more likely to be affected by hypertensive disorders in pregnancy (7.9% *vs*. 3.3%). There were no significant differences in maternal height, smoking or alcohol consumption during pregnancy between the two groups. Among mothers with GDM and information available on treatments (n=243), 24 (9.9%) mothers required the use of insulin in the management of hyperglycemia, the rest took dietary and life style interventions only. The infants of mothers with GDM had a slightly shorter average gestational age at delivery (mean: 38.8 *vs*. 39.0 weeks), and were more likely to be delivered via cesarean section (59.5% *vs*. 50.5%), while birth weights were comparable. A slightly higher proportion of infants of GDM mothers had breastfeeding less than 6 months (29.6% *vs*. 23.7%).

**Table 1:** Characteristics of mothers and children under study in the Shanghai Birth Cohort.

	All (n=2055)	GDM (n=369)	Euglycemic (n=1686)	P
Mothers				
Age (at delivery), years	29.0 ± 3.8	30.2 ± 4.1	28.7 ± 3.7	**<0.001**
Ethnicity, Han (%)	1765 (98.7)	264 (98.1)	1501 (98.8)	0.61
Education, university (%)	1167 (56.9)	167 (45.4)	1000 (59.4)	**<0.001**
Primiparity (%)	1532 (85.8)	224 (83.9)	1308 (86.1)	0.39
Height, cm	162.1 ± 5.0	161.9 ± 4.8	162.1 ± 5.1	0.50
Pre-pregnancy weight, kg	59.2 ± 9.6	63.4 ± 11.1	58.3 ± 9.1	**<0.001**
Pre-pregnancy BMI, kg/m2	22.5 ± 3.4	24.1 ± 3.9	22.2 ± 3.1	**<0.001**
Category				**<0.001**
Underweight (<18.5)	162 (8.0)	17 (4.7)	145 (8.7)	
Normal weight (18.5-24.9)	1449 (71.5)	212 (58.6)	1237 (74.3)	
Overweight/obesity (25.0-29.9)	355 (17.5)	106 (29.3)	249 (15.0)	
Obesity (≥30.0)	61 (3.0)	27 (7.5)	34 (2.0)	
Gestational weight gain, kg	12.4 ± 4.1	9.7 ± 4.1	13.0 ± 3.9	**<0.001**
Category				**<0.001**
Inadequate (%)	650 (33.1)	180 (50.8)	470 (29.2)	
Adequate (%)	871 (44.4)	129 (36.4)	742 (46.1)	
Excessive (%)	442 (22.5)	45 (12.7)	397 (24.7)	
Hypertension in pregnancy (%)	85 (4.1)	29 (7.9)	56 (3.3)	**<0.001**
Chronic hypertension (%)	28 (1.4)	13 (3.5)	15 (0.9)	**<0.001**
Gestational hypertension (%)	57 (2.8)	16 (4.3)	41 (2.4)	0.07
Smoking in pregnancy (%)	39 (2.1)	5 (1.8)	34 (2.2)	0.87
Alcohol in pregnancy (%)	239 (13.1)	43 (15.7)	196 (12.7)	0.21
Infants				
Sex, male (%)	1086 (52.8)	195 (52.8)	891 (52.8)	1.00
Cesarean delivery, n (%)	1016 (51.3)	213 (59.5)	818 (50.5)	**<0.001**
Gestational week at delivery	39.0 ± 1.3	38.8 ± 1.2	39.0 ± 1.3	**<0.001**
Preterm birth (<37 weeks)	79 (3.8)	19 (5.2)	60 (3.6)	0.20
Birth weight, g	3401.2 ± 450.8	3389.3 ± 487.7	3403.9 ± 442.4	0.60
Breast feeding				0.06
No	44 (2.2)	7 (2.0)	37 (2.2)	
Less than 6 months	498 (24.8)	107 (29.6)	391 (23.7)	
More than 6 months	1468 (73.0)	247 (68.4)	1221 (74.0)	

Data presented are mean ± SD for continuous variables and n (%) for categorical variables. Gestational weight gain was classified as inadequate, adequate, and excessive if it was below, within, or above the recommendations of the 2009 Institute of Medicine guidelines (36). Missing data were 266 (12.9%) for maternal ethnicity, 269 (13.1%) for parity, 224 (10.9%) for smoking during pregnancy, and 237 (11.5%) for alcohol in pregnancy, 92 (4.5%) for weight gain during pregnancy, and <4% for other variables.

P values in t-tests for differences in means (for continuous variables) or Chi square tests for differences in proportions (categorical variables) between the two groups. GDM, gestational diabetes mellitus; BMI, body mass index. P values in bold: P < 0.05.

### Length/height from birth to age 4 years

**Table 2** presents the data on length/height in centimeter and for age z scores (LAZ/HAZ) comparing the offspring of GDM *vs*. euglycemic mothers. Average birth length was similar, but average LAZ/HAZ scores were consistently lower in the offspring of GDM mothers in all postnatal follow-up age points (6 weeks, 6 months, 1, 2 and 4 years), and statistically significantly so at 6 weeks, 6 months and 2 years. On average, crude LAZ/HAZ values were 0.08 to 0.22 lower in the offspring of GDM *vs*. euglycemic mothers at 6 weeks to 4 years of age. The mean differences in Z scores between the two groups showed a narrowing trend over increasing ages (Z score differences: 6 weeks -0.22, 6 months -0.20, 1 year -0.11, 2 years -0.17, 4 years -0.08).

**Table 2:** Length/height from birth to 4 years of age in the offspring of GDM and euglycemic mothers.

	All	GDM	Euglycemia	P
	Mean ± SD	Mean ± SD	Mean ± SD	
**Birth (cm)**	49.9 ± 1.5	49.8 ± 1.4	49.9 ± 1.5	0.52
LAZ score	-0.09 ± 0.81	-0.12 ± 0.75	-0.09 ± 0.82	0.51
**6 weeks (cm)**	56.6 ± 2.3	56.3 ± 2.3	56.7 ± 2.3	**0.01**
LAZ score	0.37 ± 1.14	0.19 ± 1.19	0.41 ± 1.13	**0.003**
**6 months (cm)**	68.5 ± 2.6	67.9 ± 2.5	68.6 ± 2.7	**<0.001**
LAZ score	0.53 ± 1.05	0.37 ± 1.06	0.57 ± 1.05	**0.002**
**1 year (cm)**	76.3 ± 2.6	76.2 ± 2.6	76.3 ± 2.6	0.24
LAZ score	0.45 ± 1.00	0.36 ± 0.99	0.47 ± 1.00	0.08
**2 years (cm)**	88.7 ± 3.2	88.2 ± 3.4	88.8 ± 3.2	**0.009**
LAZ score	0.57 ± 0.98	0.43 ± 1.01	0.60 ± 0.98	**0.006**
**4 years (cm)**	108.4 ± 4.8	107.9 ± 4.7	108.5 ± 4.8	**0.04**
HAZ score	0.51 ± 0.96	0.45 ± 0.93	0.53 ± 0.97	0.15

Data presented are mean ± SD. The length/height-for-age Z scores (LAZ/HAZ) were calculated according to the World Health Organization (WHO) growth references (37).

GDM, gestational diabetes mellitus; LAZ, Z scores for length -for-age; HAZ, Z scores for height -for-age.

P values in t-tests for differences between the two groups.

P values in bold: P < 0.05 for comparisons between GDM and euglycemic groups.

Adjusting for maternal and child characteristics, the offspring of mothers with GDM had consistently lower LAZ/HAZ than the offspring of euglycemic mothers, with mean differences (regression coefficients) in the range of -0.11 to -0.18 between ages 6 weeks and 4 years (adjusted P<0.05 at all the five age points: 6 weeks, 6 months, 1, 2 and 4 years) (**Table 3**). Accounting for within-subject correlations in length//height, the children born to mothers with GDM exhibited a significantly reduced linear growth from birth to age 4 years overall, with a mean linear growth deficit of 0.12 (95% CI: 0.04, 0.21) in LAZ/HAZ after adjusting for maternal height, pre-pregnancy BMI, gestational weight gain, alcohol drinking status in pregnancy, gestational age at delivery and child’s sex (other maternal and child factors did not affect the comparisons and were excluded at P>0.2).

**Table 3:** The associations between GDM and longitudinal linear growth from birth to age 4 years in the offspring.

LAZ/HAZ	Crude		Adjusted*	
	β (95%CI)	P	β (95%CI)	P
Birth (LAZ)	-0.03 (-0.13, 0.07)	0.53	0.01 (-0.09, 0.11)	0.82
6 weeks (LAZ)	-0.22 (-0.35, -0.08)	**0.002**	-0.17 (-0.30, -0.04)	**0.01**
6 months (LAZ)	-0.20 (-0.33, -0.08)	**0.002**	-0.14 (-0.27, -0.01)	**0.03**
1 year (LAZ)	-0.11 (-0.23, 0.01)	0.08	-0.13 (-0.25, -0.01)	**0.03**
2 years (LAZ)	-0.17 (-0.29, -0.05)	**0.005**	-0.18 (-0.29, -0.06)	**0.003**
4 years (HAZ)	-0.08 (-0.19, 0.03)	0.16	-0.11 (-0.22, -0.02)	**0.02**
From birth to 4 years	-0.14 (-0.23, -0.06)	**0.001**	-0.12 (-0.21, -0.04)	**0.003**

Data (β) presented are the differences in the outcomes (LAZ/HAZ) from generalized linear models comparing the offspring of GDM *vs*. euglycemic mothers at each age point, and from a GEE model for the overall impact on linear growth trajectory from birth to age 4 years.

*Maternal height, gestational age at delivery, and infant sex were forced into all adjusted models; other co-variables (maternal age, ethnicity, parity, smoking in pregnancy, alcohol drinking in pregnancy, pre-pregnancy BMI, gestational weight gain, hypertensive disorders in pregnancy, mode of delivery and breastfeeding) were subject to a stepwise regression selection process; only co-variables with P ≤ 0.20 were retained in the final parsimonious models; the included co-variables were pre-pregnancy BMI, gestational weight gain, smoking and alcohol use in pregnancy for birth length, pre-pregnancy BMI and maternal hypertensive disorders for LAZ at 6 weeks, gestational weight gain for LAZ at 6 months, pre-pregnancy BMI for LAZ at 1 year, maternal age and pre-pregnancy BMI for LAZ at 2 and 4 years in multivariable models, and pre-pregnancy BMI, gestational weight gain and maternal hypertensive disorders for linear growth from birth to 4 years in a GEE model.

GDM, gestational diabetes mellitus; LAZ, length-for-age Z score; HAZ, height-for-age Z score; GEE, generalized estimating equation.

P values in bold: P < 0.05.

Sensitivity analyses excluding gestational age, gestational weight gain, or pre-pregnancy BMI and gestational weight gain as covariates, or excluding children born to mothers with hypertensive disorders in pregnancy in the analyses showed similar results as in the main analysis (**Supplementary Tables S2**, **S3**). With further adjustment for paternal height, the associations showed a similar pattern, but could not detect a statistically significant effect of GDM on linear growth due to the relatively small sample size (data available on paternal height: n=36 for children of GDM mothers) (**Supplementary Table S4**).

Among children of mothers with GDM, there were no significant differences comparing children of mothers who received insulin treatment versus those who received dietary and lifestyle interventions only in LAZ/HAZ scores at birth (mean ± SD: -0.33 ± 0.93 *vs*. -0.06 ± 0.80, P = 0.20) or age 4 years (0.56 ± 1.02 *vs*. 0.44 ± 0.93, P = 0.60). There was no significant difference in linear growth from birth to age 4 years overall in children of GDM mothers who received insulin treatment or not (**Supplementary Table S5**).

## Discussion

In a large birth cohort with longitudinal follow-up data on measured length/height, the offspring exposed to GDM exhibited consistently shorter stature in early childhood from age 6 weeks to 4 years as compared to the offspring of euglycemic mothers, although no significant differences were observed in birth length. The finding suggests an adverse impact of GDM on linear growth in early childhood. We speculated that this could be due to “impaired” bone growth potential that could manifest shortly in early postnatal life, but towards a gradual recovery path during early childhood.

We did not detect any significant differences in birth length (although numerically lower average birth length z score in GDM), consistent with a study in northern Chinese children (16) as well as in two studies in European populations (UK and Germany) (9, 20). In contrast, some studies reported greater birth length in the offspring of mothers with GDM (8, 13). These discrepant findings could be partly due to the differences in the diagnostic criteria of GDM, and the quality of care in the management of hyperglycemia in GDM (38). We used the recent IADPSG criteria with lower glucose cutoffs than the commonly used diagnostic criteria in earlier years. Therefore, women with the diagnosed of GDM nowadays tend to have less severe hyperglycemia than those GDM diagnosed in earlier years. In the study cohort, mothers with GDM experienced less frequent excessive weight gain during pregnancy compared to mothers without diabetes, and their infants have similar birth weights as infants of euglycemic mothers, suggesting high-quality care in the management of hypoglycemia in GDM. In our study, birth length was similar in the offspring of GDM and euglycemic mothers (although numerically slightly lower in the mean birth length z score in the GDM group). However, in all subsequent follow ups during infancy, the infants of GDM mothers had consistently shorter length at ages 6 months, 6 months, 1 and 2 years. This finding is consistent with the observations in a northern Chinese study population (n=1420 infants of mothers with impaired glucose tolerance or impaired fasting glucose or newly diagnosed diabetes) at ages 3, 6, 9, and 12 months (21, 33), as well as with a study of 3-month-old infants (n=90 infants of mothers with GDM) in Canada (22). A reduced length gain in the offspring of GDM mothers was noted from 3 to 24 months in another previous study (9). These findings were corroborated in a meta-analysis encompassing 4 studies including 25,736 infants (39). However, we also noted that another study in a Chinese population did not detect any changes in LAZ trajectories in the offspring of GDM mothers (n=205) from birth to 12 months (23). Taken together, most studies suggest an adverse impact of GDM on linear growth in early postnatal life. We speculated that this could probably be due to “impaired” bone growth potential *in utero*.

There have been only a few longitudinal studies on the impact of GDM on linear growth from birth to early childhood by age 4-5 years. In the PANDORA study of children aged 0-5 years with 25% of children follow-up at age 5 years, no differences in height trajectories were detected in the offspring of GDM (n= 228) and euglycemic mothers (n=95) (25). A study in Indian children (n=41 children of mothers with diabetes) using length measurements without considering other covariates reported no differences in length/height at ages 1 and 5 years (8). In contrast, a Swedish study reported increased height at age 4-5 years in female offspring of GDM mothers (n=110) (13). These differences may be partly due to limited sample sizes and different adjustments in the analyses. In the present longitudinal study on linear growth during early childhood with the largest sample size (369 children in GDM and 1686 in euglycemic pregnancy), we have accounted for important confounders such as maternal height which may help to better delineate the longitudinal impact of GDM on linear growth.

The observed adverse impact of GDM on linear growth is consistent with two studies in adolescents. Grunnet et al. reported decreased height in the offspring of mothers with GDM (n=561) at age 9-16 years (32). Similarly, a study of Dutch children reported a slight decrease in height z score in early adolescence in the offspring of GDM (n=104) (31). Taken together, the body of evidence suggests a negative impact of GDM on linear growth from early childhood to adolescence. It remains unclear whether this may be translated into an adverse impact on adult stature.

The mechanisms underlying the negative impact of GDM on postnatal linear growth are unknown. Insulin plays a pivotal role in bone and muscle growth (40), and studies have shown that preterm infants exposed to higher glucose and insulin concentrations have shorter leg length at term corrected age (41), and shorter stature at school ages (42). A similar response might be expected in linear growth in the offspring of mothers with GDM who might have been exposed to hyperinsulinemia during fetal life. However, basic science studies have shown that insulin can directly acts on insulin receptors in the growth plate, leading to chondrocyte proliferation and differentiation (43). Insulin can regulate GH receptor expression in the liver and stimulate IGF-1 production promoting bone growth (44). Cell and animal studies indicate that the osteogenic capability of bone marrow mesenchymal stem cells could be impaired under high glucose conditions (6, 7). Hyperglycemia could activate the non-canonical Wnt/PKC pathway, increasing adipogenesis while inhibiting osteogenic differentiation, leading to reduced bone mass and impaired skeletal development (45–47). Bone marrow stem cells show signs of senescence under high glucose conditions (6, 48). Therefore, high glucose and insulin could have opposite effects on bone growth. The hyperglycemia in GDM may engender a plethora of alterations in the fetus which might impinge upon the differentiation of bone marrow mesenchymal stem cells, leading to “impaired” bone growth potential, and consequently manifested dampened linear growth in early postnatal life, although the intrauterine impact might not be severe enough for a discernable impact on birth length. However, this interpretation is largely speculative. The mechanisms remain elusive and necessitate further investigations.

Our study has limitations. Paternal height may influence linear growth in the offspring. Paternal height was missing in the majority of study subjects in the study cohort. However, we observed a similar result pattern in a sensitivity analysis incorporating paternal height. There were some differences in maternal age, pre-pregnancy BMI, GDM, child’s gestational age and mode of delivery between children who remained in *vs*. lost to follow up at age 4 years. The potential impact on the findings could not be evaluated. All study subjects were Chinese from a metropolitan city in China, more studies in other regions and populations are warranted in understanding the generalizability of the study findings.

## Conclusions

Our study data suggest that GDM may impair linear growth in early childhood in the offspring. Further studies are warranted to understand the potential long-term impact on stature and health.

## Data Availability

The raw data supporting the conclusions of this article will be made available by the authors, without undue reservation. Access to the deidentified participant research data must be approved by the research ethics board on a case-by-case basis, please contact the corresponding authors (Z-C Luo, zc.luo@utoronto.ca; Fei Li, feili@shsmu.edu.cn) for assistance in data access request.
