# Supplementary material for: Gestational diabetes mellitus and linear growth in early childhood
Source: Front Endocrinol (Lausanne). 2024 Nov 27;15:1470678. doi: 10.3389/fendo.2024.1470678 (PMC11631579; doi:10.3389/fendo.2024.1470678)
Supplement: Supplementary file 1 [file Table1.docx]

**Supplementary materials**

**Table S1.** Maternal and infant characteristics of subjects who remained in vs. lost to follow-up at age 4 years in Shanghai Birth Cohort (n=3331 singleton births)

|  | **Remained in follow-up**  **(n=2207)** | **Lost to**  **follow-Up**  **(n=1124)** | **P*** |
| --- | --- | --- | --- |
| **Mothers** |  |  |  |
| Age (at delivery), years | 29.1±3.8 | 29.5±3.7 | **0.01** |
| Ethnicity, Han (%) | 1850 (98.7) | 905 (98.3) | 0.50 |
| Education, university (%) | 1225 (55.8) | 502 (55.4) | 0.87 |
| Primiparity (%) | 1600 (85.5) | 759 (84.9) | 0.71 |
| Height, cm | 162.1±5.0 | 162.1±4.8 | 0.91 |
| Pre-pregnancy weight, kg | 59.5±9.8 | 56.4±8.8 | **<0.001** |
| Pre-pregnancy BMI, kg/m^2^ | 22.6±3.4 | 21.5±4.1 | **<0.001** |
| Category |  |  | **<0.001** |
| Underweight (<18.5) | 169 (7.7) | 163 (15.1) |  |
| Normal weight (18.5-24.9) | 1508 (68.5) | 809 (74.8) |  |
| Overweight/obesity (25.0-29.9) | 382 (17.3) | 90 (8.3) |  |
| Obesity (≥30.0) | 64 (3.5) | 23 (1.9) |  |
| Gestational weight gain, kg | 12.3±4.1 | 12.7±4.9 | 0.05 |
| Hypertension in pregnancy (%) | 87 (4.0) | 42 (5.2) | 0.14 |
| GDM (%) | 390 (18.1) | 102 (12.7) | **<0.001** |
| Smoking in pregnancy (%) | 39 (2.1) | 18 (3.2) | 0.19 |
| Alcohol in pregnancy (%) | 245 (13.1) | 91 (11.8) | 0.40 |
| **Infants** |  |  |  |
| Sex, male (%) | 1153 (52.7) | 558 (51.8) | 0.65 |
| Cesarean delivery, n (%) | 1083 (52.4) | 476 (45.6) | **<0.001** |
| Gestational week at delivery | 38.9±1.4 | 39.1±1.8 | **0.004** |
| Birth weight, g | 3387.8±472.6 | 3406.6±480.1 | 0.11 |

Data presented are mean±SD for continuous variables and n (%) for categorical variables.
*P values in t-tests for differences in means (for continuous variables) or Chi square tests for differences in proportions (categorical variables) between the two groups.
GDM, gestational diabetes mellitus; BMI, body mass index; P values in **bold**: P < 0.05.

**Table S2.** Adjusted associations between GDM and longitudinal linear growth in the offspring from birth to age 4 years in a sensitivity analysis excluding gestational age, or gestational weight gain, or pre-pregnancy BMI and gestational weight gain as covariates in step-wise regression models

| LAZ/HAZ | Model 1 | Model 2 | Model 3 |
| --- | --- | --- | --- |
| Birth (LAZ) | -0.02 (-0.12, 0.08) | -0.03 (-0.12, 0.07) | 0.01 (-0.09, 0.10) |
| 6 weeks (LAZ) | **-0.21 (-0.35, -0.07)** | **-0.18 (-0.3, -0.05)** | -0.12 (-0.25, 0.01) |
| 6 months (LAZ) | **-0.19 (-0.32, -0.05)** | **-0.20 (-0.32, -0.07)** | **-0.18 (-0.30, -0.05)** |
| 1 year (LAZ) | **-0.15 (-0.27, -0.03)** | **-0.13 (-0.25, -0.01)** | -0.09 (-0.20, 0.03) |
| 2 years (LAZ) | **-0.19 (-0.31, -0.08)** | **-0.18 (-0.29, -0.06)** | **-0.14 (-0.25, -0.02)** |
| 4 years (HAZ) | **-0.11 (-0.22, -0.004)** | **-0.11 (-0.22, -0.002)** | -0.06 (-0.17, 0.05) |
| From birth to 4 years | **-0.16 (-0.24, -0.07)** | **-0.14 (-0.22, -0.06)** | **-0.11 (-0.19, -0.03)** |

Data (β) presented are the differences in the outcomes (LAZ/HAZ) from generalized linear models comparing the offspring of GDM vs. euglycemic mothers at each age point, and from a GEE model for the overall impact on linear growth trajectory from birth to age 4 years.
* Model 1 excluded gestational age, Model 2 excluded gestational weight gain, and Model 3 excluded both gestational weight gain and pre-pregnancy BMI in stepwise regression models. Maternal height, and infant sex were forced into all adjusted models; gestational age at delivery was forced into Models 2 and 3; other co-variables (including maternal age, ethnicity, parity, smoking in pregnancy, alcohol drinking in pregnancy, pre-pregnancy BMI, gestational weight gain, hypertensive disorders in pregnancy, mode of delivery and breastfeeding) were subject to a stepwise regression selection process; only co-variables with P≤0.20 were retained in the final parsimonious models.
GDM, gestational diabetes mellitus; LAZ, length-for-age Z score; HAZ, height-for-age Z score; GEE, generalized estimating equation.
P values in **bold**: P < 0.05.

**Table S3.** Adjusted associations between GDM and longitudinal linear growth in the offspring from birth to age 4 years in a sensitivity analysis excluding children of mothers with hypertensive disorders in pregnancy in step-wise regression models

| LAZ/HAZ | ***Adjusted β (95% CI)** | **P** |
| --- | --- | --- |
| Birth (LAZ) | 0.01 (-0.09, 0.11) | 0.82 |
| 6 weeks (LAZ) | -0.18 (-0.31, -0.05) | **0.008** |
| 6 months (LAZ) | -0.14 (-0.27, -0.01) | **0.03** |
| 1 year (LAZ) | -0.13 (-0.25, -0.01) | **0.03** |
| 2 years (LAZ) | -0.18 (-0.29, -0.06) | **0.003** |
| 4 years (HAZ) | -0.12 (-0.22, -0.002) | **0.047** |
| From birth to 4 years | -0.12 (-0.21, -0.04) | **0.003** |

Data (β) presented are the differences in the outcomes (LAZ/HAZ) from generalized linear models comparing the offspring of GDM vs. euglycemic mothers at each age point, and from a GEE model for the overall impact on linear growth trajectory from birth to age 4 years.
*Maternal height, gestational age at delivery, and infant sex were forced into all adjusted models; other co-variables (including maternal age, ethnicity, parity, smoking in pregnancy, alcohol drinking in pregnancy, pre-pregnancy BMI, gestational weight gain, hypertensive disorders in pregnancy, mode of delivery and breastfeeding) were subject to a stepwise regression selection process; only co-variables with P≤0.20 were retained in the final parsimonious models.
GDM, gestational diabetes mellitus; LAZ, length-for-age Z score; HAZ, height-for-age Z score; GEE, generalized estimating equation.
P values in **bold**: P < 0.05.

**Table S4.** The associations between GDM and longitudinal linear growth in the offspring from birth to age 4 years in a sensitivity analysis including paternal height as a covariate in step-wise regression models

| LAZ/HAZ | **Crude**  **β (95% CI)** | **Adjusted***  **β (95% CI)** |
| --- | --- | --- |
| Birth | -0.08 (-0.30, 0.14) | 0.02 (-0.18, 0.23) |
| 6 weeks | -0.33 (-0.84, 0.18) | -0.09 (-0.61, 0.42) |
| 6 months | -0.13 (-0.53, 0.27) | -0.13 (-0.52, 0.26) |
| 1 year | -0.0003 (-0.36, 0.36) | 0.06 (-0.28, 0.41) |
| 2 years | -0.11 (-0.49, 0.26) | -0.09 (-0.43, 0.26) |
| 4 years | -0.01 (-0.37, 0.35) | 0.04 (-0.27, 0.35) |
| From birth to 4 years | -0.10 (-0.35, 0.16) | -0.08 (-0.32, 0.17) |

Data (β) presented are the differences in the outcomes (LAZ/HAZ) from generalized linear models comparing the offspring of GDM vs. euglycemic mothers at each age point, and from a GEE model for the overall impact on linear growth trajectory from birth to age 4 years.
*Maternal height, gestational age at delivery, and infant sex were forced into all adjusted models; other co-variables (including maternal age, ethnicity, parity, smoking in pregnancy, alcohol drinking in pregnancy, pre-pregnancy BMI, gestational weight gain, hypertensive disorders in pregnancy, mode of delivery and breastfeeding) were subject to a stepwise regression selection process; only co-variables with P≤0.20 were retained in the final parsimonious models.
GDM, gestational diabetes mellitus; LAZ, length-for-age Z score; HAZ, height-for-age Z score; GEE, generalized estimating equation.

**Table S5.** The associations between insulin treatment and longitudinal postnatal linear growth in the offspring from birth to age 4 years among children of mother with GDM

| LAZ/HAZ | **Crude**  **β (95% CI)** | **Adjusted***  **β (95% CI)** |
| --- | --- | --- |
| Birth | -0.27 (-0.64, 0.09) | -0.24 (-0.57, 0.1) |
| 1 year | -0.25 (-0.68, 0.18) | -0.28 (-0.67, 0.12) |
| 2 years | -0.22 (-0.68, 0.23) | -0.14 (-0.58, 0.3) |
| 4 years | 0.12 (-0.29, 0.53) | 0.12 (-0.27, 0.51) |
| From birth to 4 years | -0.32 (-0.66, 0.02) | -0.27 (-0.59, 0.05) |

Data (β) presented are the differences in the outcomes (LAZ/HAZ) from generalized linear models comparing the offspring of mothers treated with insulin to those treated with dietary or lifestyle interventions only at each age point, and from a GEE model for the overall impact on linear growth trajectory from birth to age 4 years.
*Maternal height, gestational age at delivery, and infant sex were forced into all adjusted models; other co-variables (maternal age, ethnicity, parity, smoking in pregnancy, alcohol drinking in pregnancy, pre-pregnancy BMI, gestational weight gain, hypertensive disorders in pregnancy, mode of delivery and breastfeeding) were subject to a stepwise regression selection process; only co-variables with P≤0.20 were retained in the final parsimonious models.
GDM, gestational diabetes mellitus; LAZ, length-for-age Z score; HAZ, height-for-age Z score; GEE, generalized estimating equation.
